# Rituximab use for lymphoplasmacytic lymphoma during continuous renal replacement therapy

**DOI:** 10.5935/0103-507X.20190040

**Published:** 2019

**Authors:** Marco Simões, Marisa Miranda, José Carda, Anália Carmo, Paulo Martins

**Affiliations:** 1 Centro Hospitalar e Universitário de Coimbra EPE - Coimbra, Portugal.

**Keywords:** Rituximab, Lymphoma, Renal replacement therapy, Renal insufficiency, Polyradiculoneuropathy, Critical care, Rituximabe, Terapia de substituição renal, Insuficiência renal, Polirradiculoneuropatia, Cuidados críticos

## Abstract

Rituximab safety and efficacy in patients with renal impairment have not been established, nor have the effects of hemodialysis on serum rituximab level. There are only a few published case reports assessing serum rituximab level pre- and postdialysis. No data have been published regarding the usage of rituximab in patients with continuous renal replacement therapy. The authors present a case of a 59-year-old female patient who presented with paraneoplastic tetraparesis. She was admitted to the intensive care unit due to alveolar hemorrhage with respiratory failure and acute kidney injury requiring continuous renal replacement therapy. After a diagnostic workup, the diagnosis of lymphoplasmacytic lymphoma was established. Therapy with rituximab and cyclophosphamide was started. Rituximab levels were determined in serum and dialysate. No rituximab was found in the dialysate. The patient died after 2 months in the intensive care unit from nosocomial pneumonia due to multidrug-resistant *Pseudomonas aeruginosa*.

## Abstract (pt)

A segurança e a eficácia do rituximabe em pacientes com comprometimento renal não foram estabelecidas, e o mesmo ocorre com os efeitos da hemodiálise nos níveis séricos de rituximabe. Atualmente, apenas alguns relatos de caso avaliaram o nível sérico de rituximabe antes e após a diálise. Não foram até aqui publicados dados relativos ao uso de rituximabe em pacientes sob terapia de substituição renal contínua. Os autores apresentam um caso referente a uma mulher com 59 anos de idade atendida com quadro de tetraparesia paraneoplásica. Ela foi admitida no serviço de medicina intensiva devido a hemorragia alveolar com insuficiência respiratória e lesão renal aguda, que necessitou da utilização de terapia de substituição renal contínua. Após os procedimentos diagnósticos, estabeleceu-se o diagnóstico de linfoma linfoplasmocítico. Deu-se início ao tratamento com rituximabe e ciclofosfamida. Os níveis de rituximabe foram determinados no soro e no dialisato. Não se encontrou qualquer nível de rituximabe no dialisato. A paciente faleceu após 2 meses no serviço de medicina intensiva por pneumonia nosocomial causada por *Pseudomonas aeruginosa* resistente a múltiplos fármacos.

## INTRODUCTION

Rituximab safety and efficacy in patients with renal impairment has not been established, nor have the effects of hemodialysis on serum rituximab level. There are only a few published case reports assessing serum rituximab level pre- and postdialysis.^(1-4)^ No data have been published regarding the usage of rituximab in patients with continuous renal replacement therapy (CRRT).

## CASE REPORT

The authors present the case of a 59-year-old female patient. She came to the Emergency Department in April 2017 with back pain, lower limb pain, dysesthesia and diminished strength on the lower limbs, which impaired orthostatism and gait. These complaints had been evolving for 5 days. The clinical examination confirmed the diminished strength in her limbs, with distal predominance accompanied by sensitive deficit and the absence of some osteotendinous reflexes. Her past medical history revealed leukocytoclastic vasculitis diagnosed in 2014, treated with dapsone 100mg i.d.

The patient was admitted with a suspected diagnosis of Guillain-Barré syndrome and was treated with i.v. immunoglobulin for 5 days. Although she had slight clinical improvement, she still had severe motor impairment. Therapy with plasmapheresis was initiated but had to be suspended due to acute facial nerve paresis. Cranial, spinal and thoraco-abdominal examinations were normal. Spinal magnetic resonance imaging revealed minor degenerative changes without medullar compression. Laboratory examination revealed slight microcytic anemia and elevation of erythrocyte sedimentation rate, angiotensin-converting enzyme, ferritin and rheumatoid factor. Tumor markers were negative. Serum protein electrophoresis revealed diminished albumin and elevated gamma fraction. She had immunofixation without a monoclonal component and negative autoimmune markers. Serologies were also negative for acute infection. Electromyography was suggestive of axonal polyneuropathy.

After one month, she was discharged home. However, she was readmitted due to worsening of motor complaints. Nerve, skin and muscle biopsy was suggestive of vasculitis. The first bone marrow examination revealed 1.5% clonal B cells with a phenotype similar to lymphoplasmacytic lymphoma (LPL).

The patient's clinical status worsened during the second hospital stay, with alveolar hemorrhage, acute kidney injury and acute respiratory failure, requiring invasive mechanical ventilation and intensive care unit (ICU) admission. On day 6 of her ICU stay, CRRT was initiated.

The diagnosis of LPL was established based on the results of diagnostic exams. Bone marrow aspirate with morphology as well as the bone marrow biopsy showed an infiltrate composed of small lymphocytes admixed with variable numbers of plasma cells and plasmacytoid lymphocytes, some of them with nuclear pseudoinclusions. Immunophenotyping by 8-color flow cytometry and the presence of the MYD88 L265P mutation helped to confirm the diagnosis. Therapy with the alkylating agent cyclophosphamide 0.75g/m^2^ every 3 weeks and the anti-CD20 monoclonal antibody rituximab (MabThera^®^) 375mg/m^2^ weekly was started on the 10th day of her ICU stay. She developed pancytopenia after treatment, requiring blood and platelet transfusion. As the patient was under CRRT, the efficacy and safety of rituximab and the ideal administration protocol were questioned. Rituximab's phase II and III trials excluded patients with renal impairment. The recommended dosage and safety for patients under hemodialysis had not been established. All published data on this subject came from sporadic case reports, in which the drug was not found in the dialysate, the therapeutic plasma levels were reached and the treatment was effective. There were no published data on the usage of rituximab during CRRT. We decided upon off-label usage of rituximab as a potential lifesaving therapy (considering the LPL diagnosis and the related neuropathy). No dosage adjustment was made (375mg/m^2^ weekly). In the second rituximab cycle, serum levels were determined before and after drug administration. No drug was found in the dialysate (Table 1). Serum anti-rituximab antibody and rituximab levels were determined in serum using the Lisa-Tracker Duo Rituximab enzyme-linked immunosorbent assay (ELISA) kit (Theradiag, France). Rituximab was considered undetectable at concentrations under 2µg/mL. The limit of detection of anti-rituximab antibodies reported by the manufacturer was 5ng/mL.

**Table 1:** Rituximab blood and dialysate levels

	Before drug infusion	After drug infusion (1 hour)	Dialysate	Laboratory reference
Rituximab (µg/mL)	43.6	72.7	< 2.5	> 25
Rituximab, Ab. (ng/mL)	< 10	< 10	< 10	< 10

Continuous renal replacement therapy was performed on a Baxter Prismaflex^®^ CRRT machine using AN69 membranes (filter reference ST150) and citrate regional anticoagulation. The filter was replaced just prior to drug infusion and 5 days after infusion due to clotting. The dialytic solution used was Phoxilium^®^. The CRRT settings used are presented in table 2.

**Table 2:** Continuous renal replacement therapy settings used

Blood flow rate	110mL/minute
Preblood pump (Citrate)	1100mL/hour
Dialysate	500mL/hour
Replacement	400mL/hour
Ultrafiltration	150mL/hour
Dialysis dose	32mL/kg/hour
Filtration fraction	28%

There was no improvement in the polyneuropathy symptoms. She died on the 65th day of the ICU stay from nosocomial multidrug-resistant *Pseudomonas aeruginosa* pneumonia.

## DISCUSSION

Lymphoplasmacytic lymphoma is a neoplasm of small B monoclonal plasmacytoid lymphocytes and plasma cells, usually with focal or diffuse bone marrow infiltration. Sometimes lymph nodes and the spleen are also involved.^(5)^ The majority (up to 95%) of LPLs have MYD88 L265P mutations, but these are absent or rare in other IgM-secreting B-cell malignancies. The new mutant protein triggers tumor growth and resistance to cell death. Although LPL is often associated with a paraprotein, usually of the IgM type, this is not required for the diagnosis and sometimes can be absent.^(6,7)^ Although patients with asymptomatic disease can maintain a watchful waiting strategy, LPL with cytopenias or symptomatic disease manifested by hyperviscosity from IgM paraprotein, amyloidosis, cryoglobulinemia, central nervous system involvement or severe and/or advancing peripheral neuropathy should be considered for therapy. For symptomatic patients with LPL, options include rituximab monotherapy or rituximab in combination with alkylating agents (bendamustine or cyclophosphamide), proteasome inhibitors (bortezomib) or nucleoside analogs (fludarabine). Rituximab alone is well suited for more indolent disease and for those for whom aggressive chemotherapy is inappropriate.^(8)^

Rituximab safety and efficacy in patients with renal impairment has not been established. In the pivotal phase III trial, relapsed or refractory low-grade or follicular non-Hodgkin lymphoma (NHL) patients with serum creatinine > 2.0mg/dL were excluded from enrollment.^(9)^ Similar exclusion criteria were applied in the 2 first-line therapy trials for low-grade or follicular NHL and in the 3 diffuse large B-cell lymphoma (DLBCL) trials. In the CLL8 trial of previously untreated patients with chronic lymphocytic leukemia (CLL), patients were required to have a creatinine clearance (CrCl) ≥ 70mL/min for enrollment.^(10)^ In the REACH trial of previously treated CLL patients, patients with a CrCl < 60mL/min were excluded from the study. No reason was presented for the exclusion of patients with renal impairment in these trials. 

The effects of hemodialysis on serum rituximab level have not been established. Published data are limited to a small study and case reports assessing serum rituximab level pre- and postdialysis in patients with renal impairment requiring dialysis.

Ochi et al. conducted a retrospective study including 8 patients treated with rituximab and chemotherapy while receiving dialysis.^(1)^ Rituximab 375mg/m^2^ (no dose reduction) was given as part of the R-CVP, R-CHOP, or R-THPCOP regimen. Patients received hemodialysis 24 hours after chemotherapy. Overall, 7 patients had a complete response, 1 patient had a partial response after a median follow-up of 20 months, 1 patient died due to lymphoma at 45 months, and 1 patient died from hepatocellular carcinoma at 18 months. All patients experienced neutropenia. Other adverse events included anemia (n = 4), thrombocytopenia (n = 3), infection (n = 3), febrile neutropenia (n = 2), and peripheral neuropathy (n = 2).

Serum rituximab level and its elimination were evaluated in a 54-year-old male with low-grade NHL and immune thrombocytopenic purpura receiving thrice-weekly hemodialysis for end-stage renal disease (ESRD) by Jillella et al.^(2)^ Rituximab 375mg/m^2^ was administered weekly for 8 cycles. There was a resolution of lymphadenopathy and normalization of platelet counts. Serum rituximab level was measured before and after each rituximab treatment. In addition, rituximab level was measured pre- and postdialysis following rituximab therapy. The results suggested that these levels were comparable to levels reported in patients with normal renal function. Rituximab was not found in the dialysate fluid. No infusion-related adverse events were observed besides one event of life-threatening hyperkalemia, probably in the context of tumor lysis syndrome.

Gupta et al. compared rituximab levels before and after dialysis in an ESRD patient on thrice-weekly hemodialysis.^(3)^ A 65-year-old patient was treated with rituximab and CHOP for DLBCL. Rituximab levels pre- and postdialysis were 128,000ng/mL and 150,000ng/mL, respectively. The higher postdialysis levels were thought to be due to hemoconcentration. Rituximab was not detected in the dialysate fluid.

Morita et al. described a 76-year-old male on hemodialysis with B-cell primary cardiac lymphoma treated with biweekly rituximab monotherapy.^(4)^ The patient received rituximab 375mg/m^2^ followed by hemodialysis 1 hour later. His serum rituximab concentration was higher compared with that of a DLBCL patient with normal renal function. The rituximab concentration was maintained at a therapeutic level during dialysis. During the 6 cycles of rituximab, no infusion-related reaction occurred, and the patient experienced complete remission. A relapse occurred 10 months after diagnosis; the patient died 4 months later from progressive disease.

## CONCLUSION

In our research, we found no published data on rituximab usage during continuous renal replacement therapy. We decided to apply an off-label usage of rituximab as a potential life-saving therapy. We cannot prove its efficacy for the treatment of this patient's lymphoplasmacytic lymphoma because she died due to nosocomial pneumonia. Nevertheless, we verified by serum and dialysate analysis that rituximab was nondialyzable using these settings. This can probably be explained by rituximab's molecular weight and the dialyzer membrane used. We cannot exclude other mechanisms that could have contributed to the removal of rituximab, such as unspecific adsorption to the tubing and dialyzer membrane. However, there was an effective increase in its serum level after drug infusion despite ongoing continuous renal replacement therapy.
